# Maxwell Fisheye Lens Based Retrodirective Array

**DOI:** 10.1038/s41598-019-52779-1

**Published:** 2019-11-07

**Authors:** Muhammad Ali Babar Abbasi, Vincent F. Fusco

**Affiliations:** Institute of Electronics, Communications and Information Technology (ECIT), Queen’s University Belfast, Belfast, UK

**Keywords:** Electrical and electronic engineering, Applied physics

## Abstract

A Maxwell fisheye lens using parallel plate index grading is presented in this study to develop a passive retrodirective antenna array. As a proof-of-concept a design frequency of 10 GHz was selected for fabrication and experiment. The design principals of the lens are discussed, which enables 85% energy flow at the drain probe (also referred to as image point) of the lens. It is shown that the image in the Maxwell fisheye lens has a point symmetry with a reverse phase, which makes it possible to realize passive retrodirective action using the lens. This arrangement is significantly more practical than previous passive retrodirective topologies due to the un-constrained number of connections to radiating elements that it can support without the need for multi-layer technology. In the realization described here, a cross-polarized microstrip patch antenna array is connected to the source and drain probes of the lens structure in order to form the retrodirective array. The strategy for selecting the optimal transmission line lengths required to connect the antennas to the lens for maximum re-radiation power is described and implemented. Experimental results for a prototype high efficiency passive retrodirective array based on the theoretical design considerations presented in this paper are reported.

## Introduction

Maxwell fisheye lenses are inhomogeneous spheres in which all the rays leaving a point P on the lens will follow circular trajectories about the centre of the lens^1^. The optical length between the source and drain points along each of the lens circular trajectories is the same. Fisheye lenses have an important property that is necessary and enough for retrodirective action, i.e. every point on the lens *P* has an image point P*, such that the imaging is a phase inversion. In this work, we demonstrate, then utilise this property. We develop a practical finite size fisheye lens using ray theory and then we estimate its performance using electromagnetic analysis. Section II of the paper describes the design method of the Maxwell fisheye lens, section III discusses the hardware realization and the results, implementation of retrodirective array is discussed in section IV while findings are concluded in section V.

## Maxwell Fisheye Lens Design Principles

Let *x*, *y*, *z* represent a point in a Cartesian coordinate system within which a ray trajectory can be written as *r*(*t*) = *x*(*t*), *y*(*t*), *z*(*t*) where *t* is the parametric representation throughout the trajectory. A set of rays in a spherically symmetric optical system can be projected into a plane without losing generality. In our case, the lens is cylindrical and lies in a disk in the *xy*-plane with the center of the lens located at *z* = 0. Here ray trajectories *r*(*t*) can be calculated using conformal mapping of the points on a sphere onto the aforementioned *xy*-plane^2^. For this case, the path length differential on the sphere is mapped as an optical length differential in the *xy*-plane. In this way, the geodesic points on the great circle of the sphere can be mapped directly to a fisheye refractive index profile. For a spherical coordinate system with *θ* = 0° defining the *xy*-plane the required mapping takes the form,

1 $$x={(\frac{1-\sin \theta }{\cos \theta })}^{2}\,\cos \,2\varphi \,{\rm{and}}\,y={(\frac{1-\sin \theta }{\cos \theta })}^{2}\,\sin \,2\varphi $$

**Figure 1:**
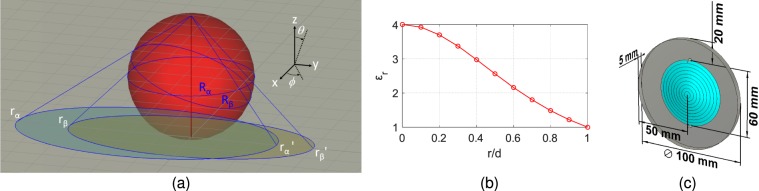
(**a**) Stereographic projection of great circles *Rα* and *Rβ* from sphere to their footprints on *xy*-plane. Great circle *Rβ* is tilted by an angle *θ*. (**b**) Theoretical permittivity profile of fisheye lens (**c**) Lens schematic when *d* = 5mm and *r* = 30 mm and *R* = 1.97*r* (top conducting sheet is removed).

This mapping is illustrated in Fig. 1(a). This is known as stereographic projection^2^ in which southern hemisphere of a sphere is mapped to the interior, while the northern hemisphere is mapped to the exterior of a circle in *xy*-plane having radius equal to the radius of the sphere. The line element segments d*x* and d*y* can be expressed in terms of spherical coordinates, and for a cylindrical lens *θ* = 0° as

2 $${n}^{2}(x,y,0)[d{x}^{2}+d{y}^{2}]={n}_{0}^{2}[d{\theta }^{2}+{\sin }^{2}\theta d{\varphi }^{2}]$$

such that at the equator, *n* is equal to the index *n*_0_ of the reference sphere. Typical lens profiles which emulate the fisheye lens^3^ in a 2D plane can be described by:

3 $$n=\sqrt{{\varepsilon }_{r}}=\frac{2{n}_{0}}{1+{r}^{2}/{R}^{2}},\,{\rm{when}}\,r\in [0,\infty )$$

where, 2*n*_0_ is the refractive index at the center of the lens, and *R* is the characteristic radius of the reference sphere.

After translating 3D fisheye characteristics into 2D, the next step is its practical realization. Generally, one can implement a variety of technologies in order to create a refractive index profile that performs fisheye focusing e.g. thin plates^4^, holy parallel plates^5^. In this paper we take a different approach in which fisheye principle is realized using the mode theory of a parallel-plate waveguide^6^. Here between ideally conducting parallel plates with spacing *d* we introduce, a medium characterized by dielectric *ε*_*r*_(*ω*) given in Fig. 1(b). The general form for the propagation constant of the fields propagating parallel to the plates is^7^,

4 $${k}_{m}=\sqrt{{k}^{2}-{m}^{2}{(\frac{\pi }{d})}^{2}}$$

when $m\in \{1,2,\cdots \}$ and $k=\omega \sqrt{{\varepsilon }_{r}}/c$. The fields will propagate as long as *k*_*m*_ is real, however when $\pi \sqrt{{\varepsilon }_{r}}/d > k$, *k*_*m*_ becomes imaginary the propagating wave decays exponentially along the direction of propagation. The parallel plate waveguide then acts as a high pass filter with cut-off frequency

5 $${\omega }_{c}=\frac{m\pi c}{d\sqrt{{\varepsilon }_{r}(\omega )}},\,{\rm{when}}\,m\in \{1,2,\cdots \}$$

**Figure 2:**
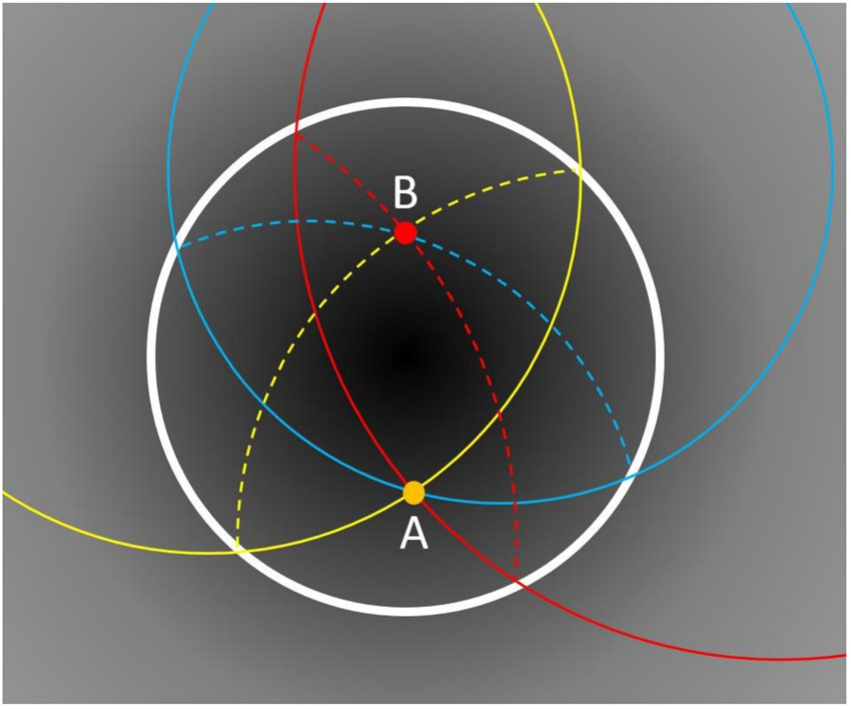
Ray tracing diagram of Maxwell fisheye lens enclosed in a spherical mirror. Points A and B represent source and image locations, respectively.

Here *m* is considered the mode number. For the propagating wave with *ω* > *ω*_c_, the phase velocity will be v = *ω*/*k*_*m*_ and the energy will transport at a group velocity *v*_*g*_ = d*ω/*d*k*_*m*_. We can control this group velocity by using a graded index profile following the fisheye Eq. (3). In this paper this is achieved by partially filling the parallel plate waveguide with dielectric material whose permittivity *ɛ*_*r*_ is fixed but whose thickness varies across the lens we can create an effective dielectric ${\varepsilon }_{r}\text{'}$ such that.

6 $${\varepsilon }_{r}\text{'}=1-t(r)(\frac{1+{\varepsilon }_{r}}{d})$$

where *r* is the radius of the parallel plates and *t* is the dielectric material thickness. Note that the notations *ɛ*_*r*_ in this paper represents general relative permittivity of a material, whereas *ɛ*_*r*_’ represents the achieved effective permittivity inside partial dielectric filled metallic parallel plates. The method of controlling the permittivity in such a case is given in^8^. Considering (3), (5) and (6) we formulated the dielectric profile between parallel plates (blue disk in Fig. 1(c)). The next step is to excite EM energy in the parallel plate region, in our case by using coax probes. An important physical limitation needs to be considered when realizing the assembly in Fig. 1(c). The approach following (3) and (6) will eventually reduce the lens profile to the near-zero-index area, which to realize a perfect fisheye operation requires the lens to be infinitely large. We side-step this by applying a circular mirror (a cylindrical reflecting metal sheet) to confine the wave propagation within a finite zone as theoretically implied in^9^. To understand how the mirror functions, consider a conformal fisheye index profile as in Fig. 2, and its multiple circular ray trajectories. For a source placed within a confined space surrounded by a mirror (white line), the incident rays (solid lines) are reflected into ray trajectories (dotted line) that focus on the image point within the now confined space.

## Fisheye Lens Development

**Figure 3:**
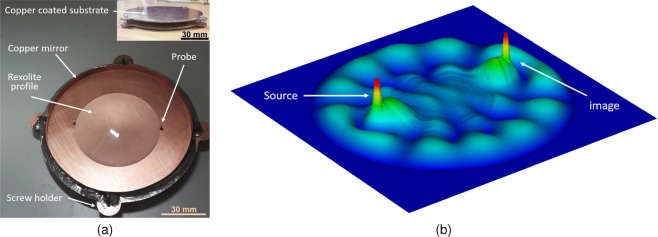
(**a**) Schematic of parallel plate Maxwell fisheye lens with 2 × coaxial probes at source and drain points. Top metallic plate is removed in the photograph (**b**) Maximum *Ez* inside the lens showing the source and image fields. (Surface plot colour map dynamic range: 0–5.5 × 103 Vm).

The fisheye lens schematic presented in Fig. 3(a) comprises of two perfectly electrically conducting, PEC, plates spaced with *d* = 5 mm. The substrate profile used to fill in the parallel plate waveguide is Rexolite^10^, *ɛ*_*r*_ = 2.53, dispersion factor = 0.00066, and low coefficient of linear thermal expansion = 3.8 × 10^−5^ 1/°F in inches. A circular band of copper of diameter = 100 mm is used to define a closed boundary for the lens, see Fig. 2. A coaxial probe with *h* = 4.3 mm (selected for 50Ω match) is used to excite the lens while the same probe length is used to form the drain point. A Triumph Dulex milling machine is used to machine the lens profile from a cylindrical Rexolite stock, and then a Tech-Gen precision finisher is deployed in order to polish the surface, making sure to have a surface roughness of <λ/8 across the lens profile. Full-wave EM simulations using CST Microwave Studio^11^ show the maximum electric fields within this confined space. Energy focusing at the image point is illustrated via the field strength plot in Fig. 3(b) confirming the operation of the device.

**Figure 4:**
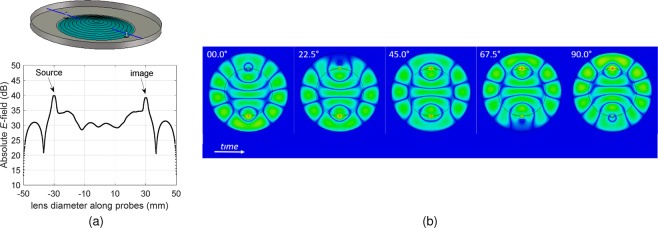
(**a**) 1D maximum absolute Ez along the blue line indicated in the lens structure placed 0.25 mm above the lens base. (**b**) Time lapse representation of real Ez inside the Maxwell fisheye lens when the Ez phase increases from 0° to 90°. Source point is at the bottom edge and image point at the top edge. (Colour map dynamic range: 0–8 × 103 Vm).

Figure 4(a) shows the focusing capabilities of the device, here ~85% simulated *E*-field can be extracted from the drain point. Since the lens structure is confined, we believe the remaining field loss is associated with imperfect imaging in the active drain located at the image point of the lens, as argued in^12,13^. A wave leaving the source in the fisheye lens follows a circular path and focuses at the image point. The real component of the electric field in time lapse images are shown in Fig. 4(b), here the difference in the propagating EM waves along the edges and centre of the lens can be clearly seen. In simulation we noted that if we locate the mirror very close to the probe location, energy loss to impedance mismatch occurs, which is undesirable for the device operation. The waves propagating between the outer edge of the lens (mirror) and the start of the Rexolite dielectric are found to have the same *v*_*g*_ which aids in image formation at the drain probe through phase alignment. Due to multiple transitions, from mirrored parallel plate, to dielectric filled parallel plate and coax-type probe (drain) it is not straight forward to identify which modes are propagating in these portions of the fisheye lens structure. The wave equation solution for two parallel plates are characterized by *m* (Eq. (5)) and TE_*m*_ and TM_*m*_ modes are defined on discrete frequencies with specific cut-off frequencies. It is important to note that a solution of wave equation with no magnetic-field in the direction of propagation (TM_*m*_) has a fundamental mode (TM_0_) that has no cut-off frequency. Considering an infinite parallel plate waveguide where wave is propagating along z-direction, at the TM_0_ mode, the propagation constant is equal to *k*. This means that the TM_0_ mode has neither electric nor magnetic field along the direction of propagation, making it a transverse electro-magnetic (quasi-TEM) mode, also supported by the lens structure.

Drain probe location in our work verifies the location identified by the time reversed scattering and transformation optics techniques^13–16^, however it is different from the optical fisheye lens in^17^. Note that the operation frequency has a direct link to the optimal drain location in a mirrored fisheye lens. Since in this work we focus only on a narrow band around 10 GHz frequency operation, further details of this relation are not emphasized here and can be found in^18^. Other ways of close-to-perfect drain from fisheye lens is with the waveguide feeds^19–21^.

**Figure 5:**
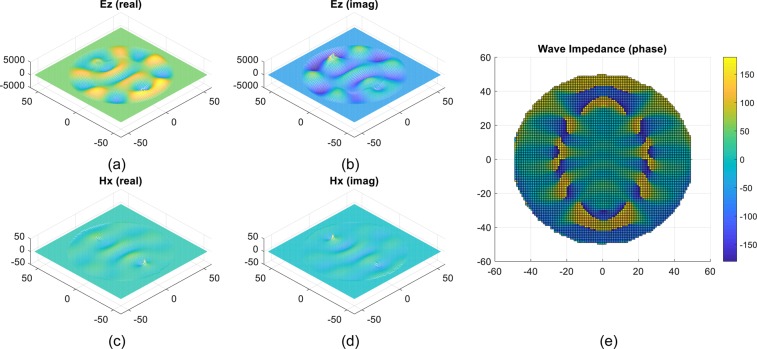
(**a**) Real and (**b**) imaginary part of the dominant component of the electric field. (**c**) Real and (**d**) imaginary part of the dominant component of the magnetic field. (**e**) Wave impedance phase 0.25 mm inside the lens structure.

To confirm the phase conjugation, we simulated *E*_*z*_ and *H*_*x*_ 2.5 mm inside the lens structure using a 2D mesh with pixel size = 1.15 × 1.15 mm^2^. The results are shown in Fig. 5(a–d). We used these results to compute the wave impedance phase as shown in Fig. 5(e). 180-degree transformation of the phase is observed when comparing the semi-circular side contain the source to the semi-circular side containing the image, confirming P* at the drain probe. The wave impedance map Fig. 5(e) allows us to choose the impedance location for the coaxial probe insertion position and its depth such that 50 Ω circuit impedance match to the external environment is ensured.

## Retrodirective Array Operation

The Van Atta array requires multiple crossovers^22,23^ hence multilayer implementation which can be lossy and are difficult to rescale for larger arrays. In the fisheye lens, when multiple sources and drain probes pairs are placed along the outer boundary of the profiled Rexolite dielectric (Fig. 3), phase conjugation analogous to Van Atta phase aligned transmission lines (e.g.^24,25^) is possible without the requirement of crossovers. Next, we show how to create a retrodirective reflector using the fisheye lens.

**Figure 6:**
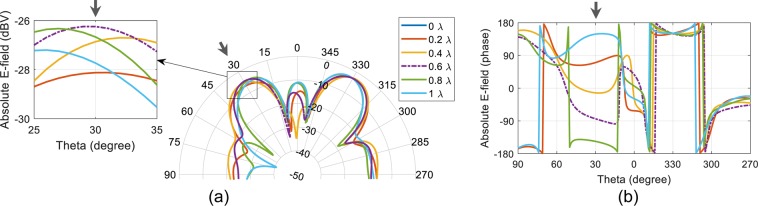
Transmission line length variation and corresponding far-field absolute *E*-field along the forward half space of the array representing the retrodirective action when DoA = 30° (**a**) Magnitude (**b**) Phase. Multiple colours are used to differentiate between closely spaced contours.

**Figure 7:**
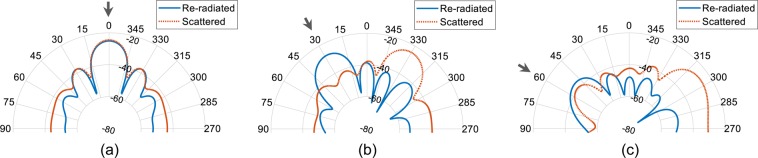
Far-field absolute *E*-field when re-radiated and scattered fields are separated numerically at (**a**) DoA = 0°, (**b**) DoA = 30° and (**c**) DoA = 60°.

**Figure 8:**
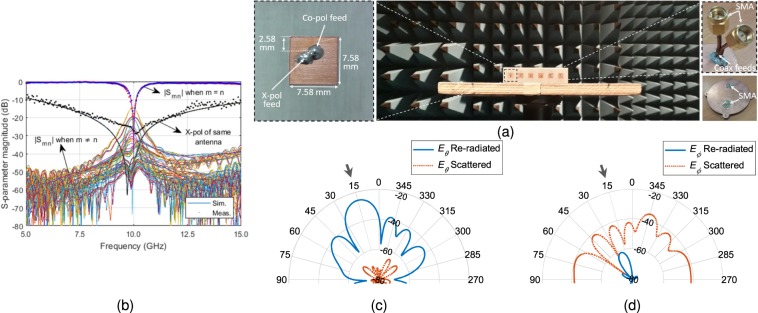
(**a**) Photograph of the 6-element antenna array in anechoic chamber, array unit cell geometry and coax feed with SMA connectors behind the antenna array to be connected to the SMA connector of the fisheye lens via standard coax cable of length l. (**b**) S-parameter representing return loss and mutual coupling between multiple probes of the array. (**c**) Far-field absolute re-radiated and scattered *E*_*θ*_-field and (**d**) *Eϕ*-field.

**Figure 9:**
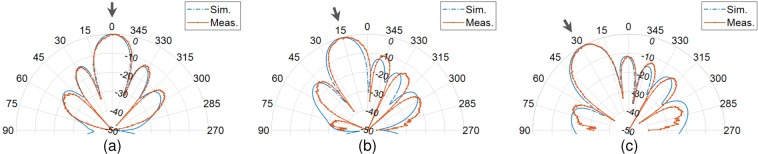
Retrodirective *E*_*θ*_-field when (**a**) DoA = 0°, (**b**) DoA = 15° and (**c**) DoA = 30°.

We first designed a λ/2 spaced 6-element microstrip patch antenna array operating at 10 GHz. The antenna unit cell used in the array is similar to a dual-feed microstrip patch antenna configuration^26^ and is optimized for 10 GHz operation. Also, the antenna used in this work have an optimized location of coax feed such that the distance of co-polarization (co-pol) and cross-polarization (x-pol) feed is same from the closest edge of the antenna. This makes the antenna structure square. The patch antenna unit cell is designed and fabricated on a 0.813 mm thick Rogers 4003 C grounded substrate (relative permittivity *ɛ*_*r*_ = 3.55 and electric loss tangent tan*δ* = 0.0027). Same unit cell is replicated to form 6-element array. Based on the incoming wave’s direction of arrival (DoA), we deduced the re-radiated and scattered patterns by qualitative and physical considerations when standard phased aligned transmission lines are replaced by the fisheye lens, e.g. Figure 6 shows the patterns when a plane incident wave at DoA = 30° hits the patch antenna array. As required for retrodirective array functionality the resulting magnitude patterns evaluated in the plane normal to the *E*-field component of the incident wave shows that there is a re-radiated portion of field toward the DoA and also a scattered field which has maximum field strength specular to the direction of the incoming wave. We find that the overall re-radiated energy is affected by the length of the transmission lines connecting the fisheye lens to the array antenna. Consider all the lens to array interconnect transmission line lengths to be equal, and

7 $$l=p\lambda +q\lambda $$

when $p\in \{0,1,2,\cdots \}$ and $0 < q < 1$. The length of a transmission line in (7) is deconstructed in two parts. First part is an integer multiple of wavelength, i.e. 1λ, 2λ, 3λ … and second is an additional section of a transmission that is subwavelength long represented by *q*. As observed in^27^ length *q* has an impact on the re-radiation and this impact is most pronounced along the DoA. Another observation is that the phase of the re-radiated field changes with a change in *q*, while it has negligible impact on the phase of the field along the direction of reflected waves (in this case about 330°). We also observed that for the microstrip patch antenna array, maximum re-radiation occurs when *q* = 0.6λ which is close to the conclusions in^27^ for a classical Van Atta configuration. Optimum transmission line length governed by *q* has an impact of approximately 1.5 dB in the re-radiated field strength along the DoA. With an optimal *q* = 0.6λ, retrodirective array performance for several representative DoA’s is shown in Fig. 7. As the DoA is skewed away from the broadside direction of the array, the re-radiated field strength weakens such that the difference between re-radiated field at DoA = 60° is ~13 dB below compared to the re-radiated field at DoA = 0°. To isolate the retro and scattered patterns we used orthogonal polarization diversity such that the co-polarization component of the antenna is connected to the source probe while the cross-polarization component is attached to drain probe. It is important to mention that mutual coupling will have an impact on the results, and in the results below this is taken into consideration. The impact is reduced by designing the antennas with low return loss (<−10 dB), high port isolation between co- and cross-polarization ports of the same antenna (i.e. < −20 dB) and minimum achievable coupling between neighbouring array elements. Fabricated microstrip patch antenna unit cell, co- and cross-polarization feed, and the 6-element array is presented in Fig. 8(a). Note that we used phase aligned coax cables with SMA type RF connectors as transmission line of length *l*. Simulated and measured results of the array are shown in Fig. 8(b). Measured |S_11_| <−10 dB bandwidth of the antenna unit cell is from 9.88 GHz to 10.11 GHz. Although the proposed fisheye lens structure is frequency dependent^18^ but since our application demanded the bandwidth limitation to <300 MHz, we consider the same limit for the fisheye lens design. Results for the wave with a DoA = 15° are presented in Fig. 8(c,d) when *E*_*θ*_ and *E*_*ϕ*_ represent the cross- and co-polarization components of the fields. Undesirable scattered fields are shown to be nullified by the proposed approach. Results evaluated by experiments shown in Fig. 9 (*E*_*θ*_ component in far-field) verifies the theoretical prediction.

The antenna unit cell number in passive retrodirective array in our work is 6, while this number is different in other retrodirective array examples in literature. Uniform linear array (ULA) configuration, for instance, are shown to have 2^28^, 4 and^29,30^ 16^31^ antenna units. In addition to azimuth direction, elevation direction are also captured using uniform rectangular array (URA) configurations, shown to have 25^32^, 32^33,34^, even 40^35^ antenna unit cells. This makes it very difficult to directly compare our approach with different retrodirective mechanisms. However, the measured *E*_*θ*_-field results in Fig. 9 show a normalized comparison with the simulated scenario, proving the proposed system to be a practically viable solution.

## Conclusion

A design approach for a cylindrically bounded Maxwell fisheye lens connected to a microstrip antenna array for retrodirective operation is presented. The operation of the lens connected to a linear array is shown to produce retrodirective action, wherein the re-radiated retrodirected beam has high isolation from un-wanted specular reflections by using polarisation diversity. Engineering details including the optimum fisheye lens design and transmission line lengths connecting the lens to the array are discussed. The approach suggests significant enhancement in the degrees-of-freedom for the implementation of high efficiency passive retrodirective array.

## Data Availability

The dataset based on measurements is available at go.qub.ac.uk/fisheye-lens.
